# The Effect of Cooling on the Degree of Crystallinity, Solid-State Properties, and Dissolution Rate of Multi-Component Hot-Melt Extruded Solid Dispersions

**DOI:** 10.3390/pharmaceutics12030212

**Published:** 2020-03-01

**Authors:** Dean Hurley, Mark Davis, Gavin M. Walker, John G. Lyons, Clement L. Higginbotham

**Affiliations:** 1 Materials Research Institute, Athlone Institute of Technology, Athlone N37 F6D7, Ireland; d.hurley@research.ait.ie (D.H.); slyons@ait.ie (J.G.L.); 2 Synthesis and Solid State Pharmaceutical Centre (SSPC), Bernal Institute, University of Limerick, Limerick V94 T9PX, Ireland; mark.davis@ul.ie (M.D.); gavin.walker@ul.ie (G.M.W.)

**Keywords:** solid dispersion, cooling, glass transition, extrusion, solubility, amorphous, crystallization

## Abstract

The effect of cooling on the degree of crystallinity, solid-state and dissolution properties of multi-component hot-melt extruded solid dispersions [SD] is of great interest for the successful formulation of amorphous SDs and is an area that is unreported, especially in the context of improving the stability of these specific systems. The thermal solid-state properties, degree of crystallinity, drug–polymer interactions, solubility and physical stability over time were investigated. X-ray powder diffraction [XRPD] and hyper differential scanning calorimetry [DSC] confirmed that indomethacin [INM] was converted to the amorphous state; however, the addition of poloxamer 407 [P407] had a significant effect on the degree of crystallinity and the solubility of the SD formulations. Spectroscopy studies identified the mechanism of interaction and solubility studies, showing a higher dissolution rate compared to amorphous and pure INM in pH 1.2 with a kinetic solubility of 20.63 µg/mL and 34.7 µg/mL after 3 and 24 h. XRPD confirmed that INM remained amorphous after 5 months stability testing in solid solutions with Poly(vinylpyrrolidone-co-vinyl acetate) [PVP VA64] and Plasdone S-630 [PL-S630]. Although cooling had a significant effect on the degree of crystallinity and on solubility of INM, the cooling method used did not have any significant effect on the amorphous stability of INM over time.

## 1. Introduction

Within both the pharmaceutical industry and academia, there is a strong interest in utilizing hot melt extrusion [HME] to prepare amorphous solid dispersions [SDs] which have the potential to significantly improve the kinetic solubility and thus, the bioavailability of BCS class II drugs which have poor aqueous solubility and high permeability [1]. The conversion of the active pharmaceutical ingredient [API] to the amorphous state increases dissolution rate and apparent solubility as the penetration of solvents is not prohibited by an endothermic barrier attributed to the disorder of crystalline lattices [2]. During dissolution the carrier traps the dispersed API in a high energy state, maintaining supersaturation in the medium over time and preventing crystallization [3].

Bravo et al. (2004) reported that HME has many significant benefits such as the elimination of solvents, good content uniformity due to the low drug loading used, dosage forms of a desired shape, including tablets, pellets and implants can be easily manufactured by this process; HME is also a continuous process and can be scaled up [4] and finally, HME allows conversion of crystalline drugs to amorphous form or dispersion of API into very small particles, enhancing bioavailability and improving patient compliance. 

However, the application of high shear inherent to the process and high processing temperatures presents a significant challenge when dealing with APIs that are thermo-sensitive. However the inclusion of plasticizers as excipients can lower the processing temperatures associated with HME and can modify the physicochemical and dissolution properties of poorly water-soluble APIs [2,5]. Understanding the physicochemical properties of both the API and polymeric carriers is important when manufacturing SDs as it is the properties of the formulation that is being extruded that dictate the dissolution behaviour.

Although the potential of amorphous SDs is enormous, the physicochemical factors of these systems need to be fully understood as they have a significant effect on the dissolution and stability of the amorphous drug. Various factors can affect the solid-state and dissolution properties of these systems and can result in undesirable behaviours, such as nucleation, phase separation and recrystallization during storage [6]. 

Previous studies have focused on how various extrusion process parameters affect the dissolution behaviour of indomethacin [INM]. For example, Liu et al. (2009) reported the effect of extrusion process parameters on the dissolution behaviour of INM in Eudragit EPO SDs. In this study, three process conditions—set mixer temperature, screw speed and residence time—were studied. The results show that the dissolution rate of INM increased with increasing screw rotating speed or the mixer set temperature. Further research was carried out using semi-crystalline polymers such as Poloxamer 407 [P407] to overcome the drawbacks associated with HME such as its high processing temperatures [5,7], as the processing temperatures can restrict the proccesing of thermally labile drugs [8]. However, the cooling method used during the extrusion process can also have a significant effect on the crystallization tendency and solubility of the API. Very little has been reported on how various cooling methods effect the degree of crystallinity, solid state properties and dissolution rate of multi-component amorphous SDs.

INM has been used as a model drug in this study which is a BCS class II API (low solubility, high permeability). It is well reported in the literature that preparing an SD of INM significantly improved the amorphous stability and solubility of INM [5]. Poly(vinylpyrrolidone-*co*-vinyl acetate) [PVP VA64] and Plasdone-S630 [PL-S630] in terms of monographs, have the same chemical structure; however, they have different solid-state properties and solubilities depending on the manufacturing process used. PVP VA64 and PL-S630 are commonly used carriers for optimizing the solubility of INM, and the effect of these specific carriers will be examined in this study. This study is also a continuation of the work carried out by Hurley et al., (2019) [6]. P407 was chosen as it is reported in literature that P407 cannot only overcome the drawbacks associated with HME, but increase the solubility of INM significantly [9]. The 30% INM drug–polymer mixtures were used as it was reported that maximum solubility of INM reported in the literature was 30 µg/mL after 72 h using 30% INM [10]. 

The relationship between cooling and the physicochemical/dissolution properties of the drug and polymeric carriers is an area that is very important in the manufacture and development of SDs. It is reported in the literature that slow cooling enhances the physical stability of amorphous INM [11]; however, Meng et al. (2015) stated that the faster the cooling rate, the lower the crystallinity of the API [12]. Therefore, in this study, the effect of various cooling processes are studied. This study was, to our knowledge, the first to report how various cooling methods affect the dissolution behaviour of INM using a semi-crystalline surfactant and how it affects the degree of crystallinity of amorphous SDs.

Since this study uses a semi-crystalline plasticizer to improve the solubility of INM, the degree of crystallinity of the semi-crystalline polymer in the SD formulations must be examined as it can affect the processing and physical properties of the API. 

## 2. Materials and Methods

### 2.1. Materials

**Figure 1:**
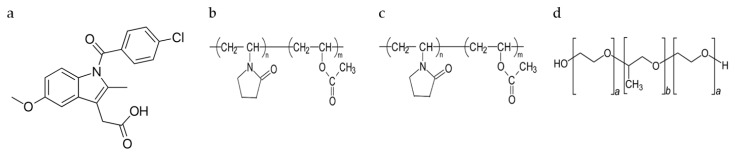
Chemical structures of (**a**) BCS class II model drug indomethacin [INM], (**b**) Poly(vinylpyrrolidone-*co*-vinyl acetate) [PVP VA64], (**c**) Plasdone S-630 [PL-S630] and (**d**) Poloxamer 407 [P407] used in this study.

Crystalline INM (1-(4-Chlorobenzoyl)-5-methoxy-2-methyl-3-indoleacetic acid), purchased from Tokyo Chemical Industry (TCI) (Oxford Science Park, Oxford, UK) (N.V.) (*M_w_* 357.79 g/mol^−1^, *T_m_* 160.0 °C *T*_g_ 42 °C) was used as the model drug [5]. INM has an aqueous solubility of 1.5 µg/mL at pH 1.2 [13]. P407, a hydrophilic non-ionic surfactant, (Mw 12,000, *T_m_* 55 °C, *T*_g_ −67 °C) and PVP VA64 (Mw 45,000, *T_g_* 107 °C, *T*_m_ 140 °C) was purchased from BASF Europe GmbH (Burgbernheim, Germany) [5]. PL-S630 (Mw 47,000, *T_g_* 106 °C, *T*_m_ 140 °C) was received as a gift from Ashland Specialties Ltd, (UK) [6]. All other chemicals and reagents were purchased from Sigma Aldrich (Wicklow, Ireland) and were of analytical reagent grade. The chemical structures of the materials used in this study are shown in Figure 1. 

### 2.2. Preparation of Physical Blends

Six different combinations of the four components were examined and 50 g of powder were used for each batch. Physical blends were mixed in a mortar and pestle for 5 min after weighing, in accordance with the drug/carrier percentages outlined in Table 1. The 30–70% INM-P407 binary mixtures were prepared as controls. Amorphous INM [aINM] was prepared by heating up to 160 °C in a stainless steel beaker using a hotplate and quench cooling using fluid nitrogen [5].

### 2.3. Hot Melt Extrusion

The compositions (%*w*/*w*) of each of the physical mixtures shown in Table 1 were extruded using a bench-top co-rotating Prism 16 mm twin-screw extruder (Thermo Fisher Scientific, Waltham, MA, USA) with a 2 mm diameter die and a length to diameter [L/D] ratio of 15/1. The physical mixtures were extruded using screws containing conveying elements and a screw speed of 100 RPM [5]. The extruder contained three heating zones which, from feeding zone to the die, had set points of 140, 160 and 180 °C. The physical blends were fed into the 15:1 L/D extruder using an automatic feeder at a *rate* of *13* ± 1 g/min. The diameter of each of the extrudates was 2mm. With the addition of the surfactant, the temperature profile was reduced by 10 °C. As the extrudates exited the die, they were air cooled to 25 °C, ground in a mortar via liquid nitrogen, and passed through a 200 µm sieve to obtain uniform sized fractions for further studies [5]. All the samples were placed in a desiccator alongside phosphorus pentoxide to remove any residual moisture.

### 2.4. Cooling Process

To investigate the crystallization tendency of the API, a selected SD formulation was air cooled [AC] to 25 °C by an air pipe connector attached to the 2 mm die of the extruder, cooled to normal room temperature (25 °C) [NT], the same as air cooling except with no air pipe connector and quench cooled using liquid nitrogen, [Liq N_2_], to examine the effect of slow and rapid cooling processes on the amorphous stability and solubility of INM. For the purpose of this study, only one SD formulation was investigated. 

### 2.5. Calculation of Solubility Parameters via Hansen Solubility Parameters (δ)

This theory was established by Hansen in 1967 when he divided the cohesion energy into 3 different components: polar, physical and hydrogen bonding forces, which all contribute to the molecular structure of any compound. This specific idea was the key aspect of Hansen solubility parameters theory [HSPs]. HSPs have been applied in the pharmaceutical industry to provide a complete understanding of the various cohesive energies within APIs and therefore, permits the prediction of their behavior under various conditions and manufacturing processes in addition to how they behave within the human body. The HSPs were calculated from their chemical structures using the combined group contribution methods of Hoftyzer and Van Krevelen, (1976), [14], according to the following Equation (1):

δ^2^ = δ^2^_d_ + δ^2^_p_ + δ^2^_h_ (1)

where

$$\delta_{d}=\frac{\Sigma F_{\mathrm{di}}}{V},\;\delta_{p}=\frac{\left(\Sigma {F_{\mathrm{pi}}}^{2}\right)}{V}\;{}^{1/2},\;\delta_{h}=\left(\frac{\Sigma F_{\mathrm{hi}}}{V}\right){}^{1/2}$$

where *i, δ, δ*_d_, *δ*_p_, and *δ*_h_*,* are the functional group within the molecule, the total solubility parameter, contribution from dispersion forces, polar interactions and hydrogen bonding, respectively. *F*_d_*i*, *F*_p_*i*, and *E*_h_*i* are the molar attraction constant due to molar dispersion forces, molar polarization forces and hydrogen bonding energy, respectively, and *V* is the volume within the lattice site [15]. 

Hildebrand and Scott (1950) developed the drug–polymer interaction parameter, χ, which can be calculated via the HSPs [16]. 

This is expressed as follows:

(2) $$\chi =\frac{Vo}{RT}\;{\left(\delta_{\mathrm{drug}}-\delta_{\mathrm{polymer}}\right)}^{2}$$

where *V*o is the volume of the lattice site, *R* is the gas constant and *T* is the absolute temperature. 

### 2.6. Attenuated Total Reflectance-Fourier Transform Infrared [ATR-FTIR] Spectroscopy

ATR-FTIR spectra of all SD formulations were analyzed via a Spectrum One, Perkin Elmer apparatus equipped with an ATR sampling universal accessory. The spectra were collected within the range of 4000–420cm^−1^, using a 16 scan per sample sequence. The compression force used was 80N, which is fixed and universal. The analysis of the ATR-FTIR spectra was performed using Perkin Elmer Spectrum software [5].

### 2.7. Raman Spectroscopy

Raman spectra of all SD formulations were attained using a Renishaw invia Raman confocal microscope (Renishaw Instruments, Gloucestershire, UK) which was attached to a motorized stage. A 785 nm laser was used to provide Raman scattered light, the laser operated at 300 mW with a laser power of 100%. The Raman Spectra of each formulation were within the range of 100 and 3200 cm^−1^, and with an acquisition time of 10 s. A ×20 lens was used and had a spot size of 50 µm [6]. A 1200 l/mm (633/780) beam path grating was used. 

### 2.8. X-ray Powder Diffraction [XRPD]

XRPD spectra of all SD formulations were attained using a Philips PANalytical Empyrean X’Pert MPD Pro using a sample spinner PW3064. Each SD formulation once ground were positioned on silica disks to provide a zero-background [5]. The diffraction pattern was within the range of 5° to 40° (2θ) with a 0.0167° step size. The total collection time was 29.845 s, using a sample rotation of 15 rpm via a PANalytical Data Collector, version 2.0. A Cu Kα (λ = 1.5418 Å) source was used, using an accelerating voltage of 40 Kv and a 35 mA anode current. A ¼” fixed divergence slit and a nickel filter, which was 0.020 mm in size, were used [5]. 

### 2.9. Hyper Differential Scanning Calorimetry [DSC]

Hyper DSC was carried out on all SD formulations using a PerkinElmer DSC 8500 that was attached to a cooling accessory which was refrigerated (PerkinElmer, UK). The purge gas used was Helium at 30 mL/min. The DSC instrument was calibrated using a heating rate of 100 °C/min using high-purity indium in order to standardize the heat flow signal and temperature [5]. Then, 2.5 mg samples were weighed and then placed in crimped DSC pans. Samples were ramped from −10 to 180 °C at 100 °C/min. The DSC analysis was performed using a PerkinElmer pyris thermal analysis software, version 10.1 and any numerical values reported are the mean ± SD of three independently prepared samples [5]. The degree of crystallinity of P407 was calculated using Equation (3):

(3) $$\chi_{c}=\frac{\Delta H_{m}}{\Delta {H^{{}^{\circ}}}_{m}\;(w)}\;100\%$$

where *χ*_c_ is the degree of crystallinity, ΔH_m_° the heat of fusion of the 100% crystalline materials, ΔH_m_ is the heat of fusion of the sample and w is the weight fraction. The value of the heat of fusion of 100% crystalline material ΔH_m_° was calculated using DSC. 

### 2.10. Preparation of pH Buffer 1.2

An amount of 2 g of sodium chloride [NaCl] was added to 500 mL of deionized water, and 8.3 mL of 0.1 M HCL was pipetted and diluted to 1 litre with deionized water. 

### 2.11. Solubility Studies

Solubility studies of the SD formulations were carried out in triplicate via the method reported by Higuchi and Connors in pH buffer 1.2 [17]. pH buffer 1.2 was prepared. A specific amount of SD formulation was added to 10 ml volumetric flasks. The suspensions were maintained at 37 °C for 24 h. This timeframe was reported in the literature to be an appropriate timeframe to achieve equilibrium [5]. Two millilitres were filtered and withdrawn through 25 mm Millex—Polytetrafluoroethylene [PTFE] syringe filters (Merck Millipore LTD, Ireland) LCR code. The syringe filters were 0.45 µm in size and hydrophilic in nature. Once diluted, the filtrates were examined for dissolved INM spectrophotometrically at 320 nm [5]. 

### 2.12. In-Vitro Dissolution Studies

The dissolution rate and supersaturation parameter [SP] of INM from SDs and physical blends was tested under non-sink conditions using the United States Pharmacopeia [USP] dissolution testing apparatus 1 (basket technique) (Distek 50947, USA) with a paddle speed of 50 rpm. The non-sink dissolution test was performed using 900 mL of pH buffer 1.2 at a temperature of 37 ± 0.5 °C. 

A formulation (in powder form) which was equal to 100 mg of INM in physical blends and SD formulations was placed within the dissolution medium. At set time points, 5 mL aliquots were withdrawn and separated through a 25mm millex-LCR PTFE hydrophilic syringe channel (0.45 µm, Merck Millipore LTD, Ireland). At each time point, a similar volume of new medium was replaced as withdrawn. The INM concentration in each example was examined utilizing a UV-1280 UV–Vis spectrophotometer (Shimadzu, Japan) and a standard curve. Pure and amorphous INM were used as controls. The INM concentration dissolved for each formulation (*n* = 3) was plotted as a function of dissolution time information being expressed as the mean ± standard deviation of duplicate absorbance estimations. The ability of the polymeric carriers to prolong and maintain INM supersaturation in pH buffer 1.2 was qualified by using the SP as reported by Chen et al. (2015) [18].

### 2.13. Statistical Analysis

Dissolution profiles of all the SD formulations using an analysis of variance [ANOVA] statistical test were compared. The area under the curve [AUC] was statistically examined to examine the effect of the amorphous form (GraphPad Prism version 5.03 for Windows, La Jolla, San Diego, CA, USA). Tukey’s Multiple Comparison post-hoc test was used to compare the means. A significance level of * *p* < 0.05 was used to signify the significance for all formulations [5]. 

### 2.14. Accelerated Amorphous Stability Studies

Accelerated stability studies were carried out under accelerated conditions (40 °C, 75% relative humidity) by placing SDs in open class vials which were stored inside a desiccator containing a saturated solution of sodium chloride to produce a relative humidity of 75%. The relative humidity inside the desiccator was checked constantly utilizing a thermohygrometer [5]. The amorphous state of the stored SDs was examined using hyper DSC and XRPD, as described previously.

## 3. Results and Discussion

### 3.1. Drug-polymer Miscibility Studies. 

#### 3.1.1. Pre-Formulation Studies

Gaikwad et al. (2017) reported that within the pharmaceutical industry, HSPs are commonly used to determine the miscibility of an API with pharmaceutical excipients/carriers in SDs. It has also been reported that HSPs have been used within the pharmaceutical industry to determine the compatibility of pharmaceutical materials, and are therefore recommended in pre-formulation and in the development of SDs and tablets [19].

Maniruzzaman et al. (2015) used the Flory–Huggins (F–H) lattice-based theory to explain intermolecular interactions between polymer and API is limited as it does not take into consideration the various multiple interactions in drug-polymer systems [20]. Therefore, the HSPs developed by Van Krevelen and Hoftyzer group were used as a substitute for the Flory–Huggins theory to explore the nature of interactions that occur within drug–polymer systems and is mainly based upon the chemical structures of each of the pure components. 

For an intermolecular interaction to occur between an API and polymer, the change in the free energy of mixing must be negative according to the laws of thermodynamics of mixing. This specific change in the free energy of mixing is related to enthalpy and entropy contributions according to the following equation (Equation (4)):

Δ*G_mix_* = Δ*H_mix_* −*T x* Δ*S_mix_* (4)

where Δ*G_mix_*, Δ*H_mix_*, Δ*S_mix_* and *T* are the Gibbs free energy, enthalpy of mixing, entropy of mixing and absolute temperature, respectively. The free energy change is spontaneous as a result of the increase in entropy of mixing and is therefore negative [20]. However, the presence of repulsive and cohesive intermolecular and intramolecular forces (e.g., dispersion force, hydrogen bonding and dipole–dipole interaction) which are present within the amorphous SD, making the intermolecular interaction between drug and polymer more complex.

The drug–polymer interaction factor was calculated via the Hildebrand-Scott method which is a hypothetical method used to calculate F–H interaction parameters [16]. It is well reported in the literature that unfavourable intermolecular interactions will result in phase separation and recrystallization if Δδ > 7 MPa^1/2^, whilst favourable intermolecular interactions and a uniform phase will occur if Δδ < 7 MPa^1/2^ [21]. 

This is also the case for drug–polymer interactions if the value for χ is close to zero, as shown in Table 2. Δδ and χ between INM and the polymers was less than 7 MPa^1/2^ and close to zero respectively, therefore they are likely to be miscible [16]. 

The molar attraction constant for INM was high (10.37 MPa^1/2^) and was similar to the values calculated for the polymers (PVP VA64: 11.86 MPa^1/2^ and PL-S630: 11.86 MPa^1/2^) due to the interaction via hydrogen bonding. The molar attraction constant values are vital for intermolecular interactions between drug and polymer to occur during the transformation to the amorphous state. See Table 3, Table 4, Table 5 and Table 6 for HSPs calculations used to predict drug–polymer miscibility and drug–polymer interaction factor (χ). 

Maniruzzaman et al. (2015) prepared amorphous solid dispersions using hot-melt extrusion using the BCS class II drug Verapamil HCL, which had a molar attraction constant of 6.95 MPa^1/2^ and reported that due to the high molar attraction constants of the APIs and polymeric carriers, they were able to contribute to hydrogen bonding [20]. As the HSPs are only theoretical, drug–polymer miscibility was further examined using various characterization, thermal analysis and solubility techniques to determine if the drug–polymer mixtures were miscible. In order for a drug and polymer to be miscible it requires the drug to be successfully converted to the amorphous form and to interact with the polymeric carrier. Conversion to the amorphous state was determined using XRPD and hyper DSC to not only determine if the drug is amorphous, but also to determine if only one glass transition temperature (*T*_g_) is present in the SD formulations. A single *T*_g_ means successful integration of the drug into the polymer. Interaction between drug and polymer was examined using ATR-FTIR and Raman spectroscopy. 

#### 3.1.2. ATR-FTIR and Raman Studies 

**Figure 2:**
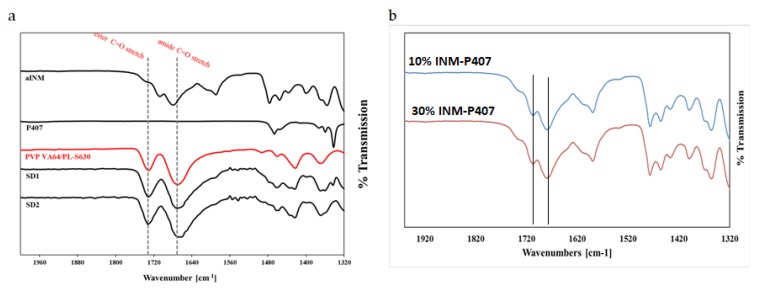
(**a**) ATR-FTIR Spectra of pure components and selected SD formulations (30% INM) showing a shift in the amide carbonyl C=O from 1672 cm^−1^ to 1680 cm^−1^. There is no shift in the vinyl acetate carbonyl peak as a result of its weaker hydrogen bond potential. (**b**) ATR-FTIR spectra of binary SDs (controls) of 10% and 30% INM-P407 drug–polymer mixtures.

ATR-FTIR was used in conjunction with Raman studies to elucidate the mechanism of interaction between INM and each of the polymers which is necessary for drug–polymer miscibility. XRPD and hyper DSC was used to confirm the amorphous nature of INM in each of the SD formulations and to determine *T*_g_. PVP VA64 and PL-S630 showed two strong bands at 1731 cm^−1^ and 1672 cm^−1^ which correspond to the C=O stretch of the vinyl acetate and the C=O stretch of the amide carbonyl respectively. When INM was converted to the amorphous form via hot melt extrusion, the amide carbonyl C=O of PVP VA64 and PL-S630 shifted to 1680 cm^−1^. This indicates that hydrogen bonding had taken place between the amide carbonyl of the polymer and the free C=O bond of the carboxylic acid of INM [5]. This was the case for all SD formulations, as shown in Figure 2a. 

**Figure 3:**
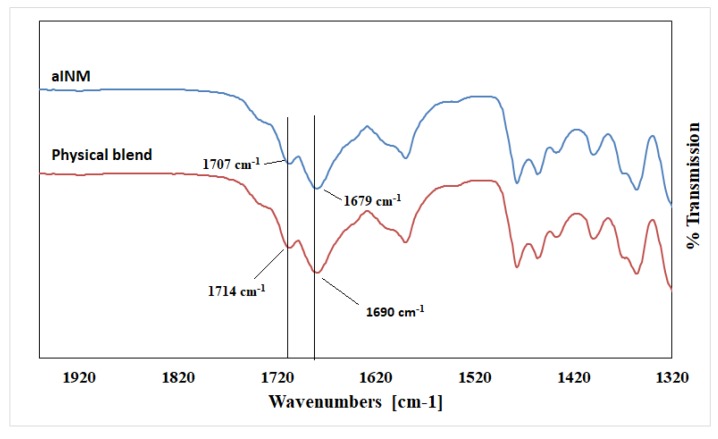
ATR-FTIR Spectra of aINM and Physical blend. Crystalline INM is still present in the physical blend as expected.

There was no change in the vinyl acetate C=O carbonyl due to weaker hydrogen bonding potential of the vinyl acetate carbonyl as reported by Yuan et al. (2015) as shown in Figure 2b [22]. Any P407 peaks within the pure P407 sample were completely absent in the ATR-FTIR spectra of the SD formulations as shown in Figure 2a and Figure 3. This indicates that P407 possibly has no intermolecular interaction with INM at the molecular level [9]. 

**Figure 4:**
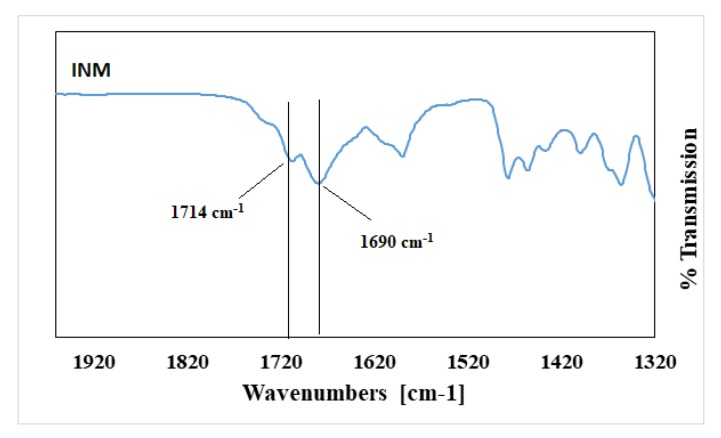
ATR-FTIR Spectrum of crystalline INM.

Pure INM contains two strong non-hydrogen bonding C=O bands at 1690 cm^-1^ (acid-acid dimer C=O stretch) and 1714 cm^−1^ (free C=O of carboxylic acid) respectively [23]. The hydrogen-bonded O–H stretch of the acid, shown in Figure 4, is overlaid on the C–H sharp stretches, as literature has reported that the free carboxylic acid O–H stretch as a result of hydrogen bonding can exist as dimers [23]. It is also important to note that in the ATR-FTIR reference spectrum of aINM, the two C=O bands at 1714 cm^−1^ (free C=O of carboxylic acid) and 1690 cm^−1^ (acid-acid dimer C=O stretch) shifted to 1707 cm^−1^ and 1679 cm^−^^1^, respectively, due to transformation to its amorphous form [5], as shown in Figure 2a, therefore do not align with the polymer peaks. The physical blends contained crystalline INM as expected as shown in Figure 3 [5]. 

Pharmaceutical drugs which are usually conjugated compounds have very strong Raman signals, while pharmaceutical excipients tend to have Raman signals which are weak [8]. Like ATR-FTIR, Raman spectroscopy can identify potential mechanism of intermolecular interaction among the polymeric carriers and INM due to a shift in the PVP VA64 and PL-S630. 

**Figure 5:**
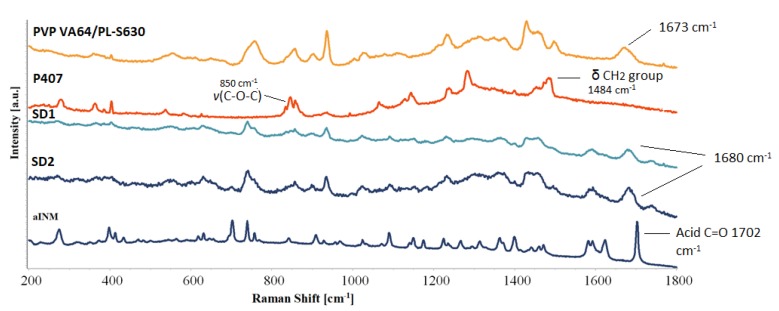
Raman spectra of crystalline INM, polymeric carriers and chosen SD formulations (30% INM), which shows interaction via hydrogen bonding as a result of the shift in the amide C=O carbonyl stretch of PL-S630 and PVP VA64. The low intense peak at 1732.00 cm^−1^ corresponds to the vinyl acetate C=O carbonyl.

Hydrogen bonding was the predicted mechanism of interaction due to polarity of INM and polymeric carriers as a result of the relatively high INM molar attraction constant calculated using the HSPs. The amide carbonyl (*v*C=O) peak at 1673 cm^−1^ shifted to 1680 cm^−1^ in all SD formulations due to hydrogen bond interaction with the –OH carboxylic acid of INM, as shown in Figure 5. The intensity of the vinyl acetate carbonyl group is weak and appears at 1745 cm^-1^ due to the weaker hydrogen bonding potential as reported by Yuan et al. (2015) [22]. 

The acid *v*C=O present at 1702 cm^−^^1^ (free C=O of carboxylic acid) of INM disappears in the Raman spectra of the quaternary and ternary SD formulations due to the low intensity of INM. Based on the Raman spectra in Figure 5, the peaks identified in the Raman spectra of aINM were not present in the Raman spectra of the amorphous SD formulations. The broad peak at 1482 cm^−1^ in the Raman spectrum of solid-state P407 reflected the deformation vibration of the δCH_2_ group. 

The *v*(O–H) of P407 was present at 3200 cm^−1^ and *v*(C-O-C) was present at 850 cm^−1^. Careful analysis of the SD formulations indicates that the bands associated with P407 were not present in the SD formulations due to the lack of an intermolecular interaction. Raman spectroscopy similar to ATR-FTIR spectroscopy identified that hydrogen bonding occurred between PVP VA64/PL-S630 regardless of the cooling method used. 

#### 3.1.3. XRPD and Hyper DSC Studies 

**Figure 6:**
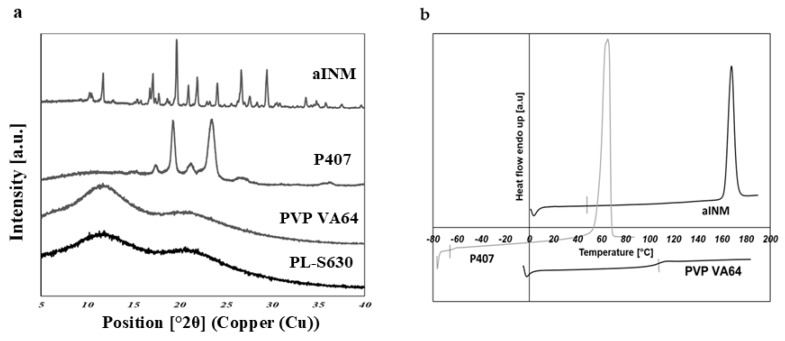
(**a**) XRPD diffractograms of the pure components and (**b**) hyper DSC thermograms of pure components after heating at 100 °C/min to 180 °C. INM is transformed to the amorphous form. (**b**) Hyper DSC shows that pure P407 displays an endothermic peak at 57.13 °C due to its semi-crystalline nature. Glass transition temperature [*T*_g_] is indicated by a tick.

**Figure 7:**
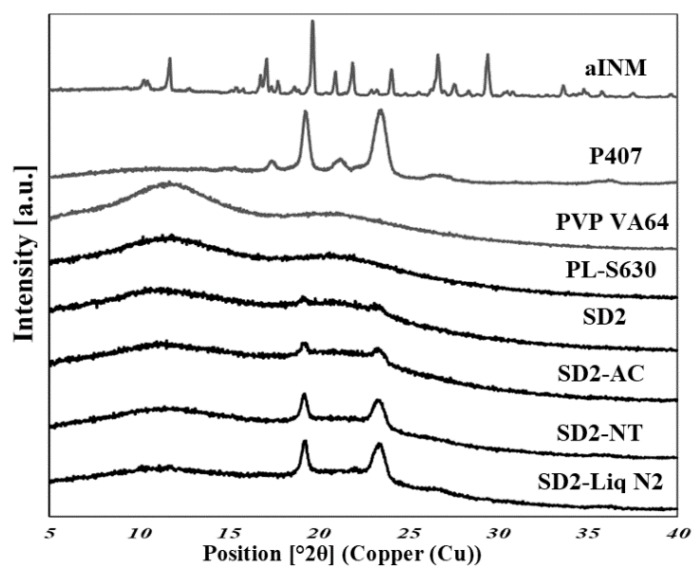
XRPD diffractograms of SD formulations and XRPD diffractograms of the SD formulation used to investigate the effect of cooling. P407 was still present in its crystalline form in all formulations (AC = Air cooled, NT = Normal room temperature and Liq N_2_ = Liquid Nitrogen).

XRPD was used to examine the amorphous nature of the drug within the SD formulations. XRPD was performed on a PANalytical Empyrean X-ray diffractometer attached to a computer running High Score Plus to collect and process data. The XRPD of INM shows multiple Bragg peaks similar to the literature values reported for INM [8], indicating the crystalline nature of the drug, as shown in Figure 6a. Figure 6a also shows the XRPD diffractograms of P407, PL-S630 and PVP VA64. Figure 7 shows the XRPD diffractograms of the SD formulations used to investigate the effect of cooling. 

The XRPD diffractograms of PL-S630 and PVP VA64 showed that they were amorphous in nature due to slight amorphous halo raised above the baseline. The presence of two Bragg peaks of P407 at 19 and 24° respectively indicates that P407 was not converted to the amorphous form as reported by Hurley et al. (2018) [5]. The drug peaks associated with crystalline INM were completely absent in the melt extrudates with various drug–polymer ratios, indicating that INM was successfully converted to its amorphous form as shown in Figure 7. Hyper DSC, like the XRPD diffractograms, also confirmed that INM was successfully converted to its amorphous form and P407 remained semi-crystalline. 

**Figure 8:**
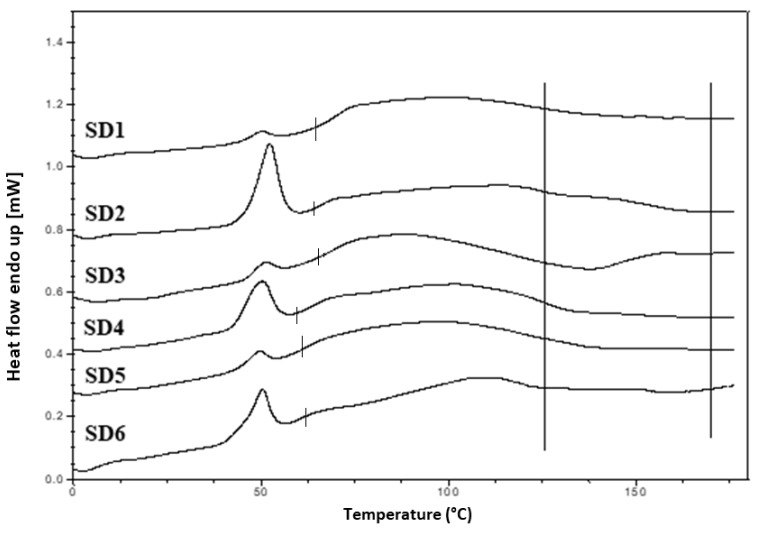
Hyper DSC thermograms of air-cooled SD formulations. Hyper DSC indicates that INM was converted to its amorphous form. The area of interest for *T*_g_ is marked by the straight lines.

**Figure 9:**
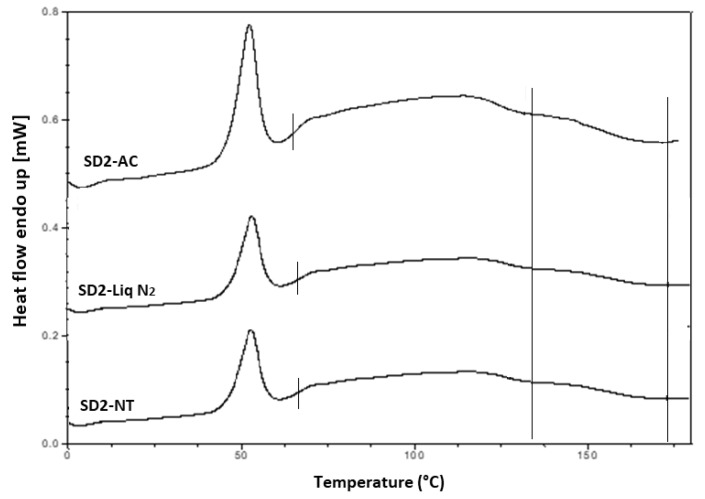
Hyper DSC thermograms of selected SD formulations used to investigate the effect of cooling. P407 was still present in its crystalline form in all formulations (AC = Air cooled, NT = Normal room temperature and Liq N_2_ = Liquid Nitrogen). Hyper DSC indicates that INM was converted to its amorphous form. The area of interest for *T*_g_ is marked by straight lines.

Hyper DSC was performed to characterize the solid state of the SD formulations and to determine the *T*_g_ of the SD formulations. The DSC thermograms of the pure components and SD formulations are shown in Figure 6b, Figure 8 and Figure 9. The % crystallinity of P407 for each of the samples was also determined. 

The thermogram of crystalline INM showed an endothermic peak at 165.47 °C corresponding to the melting endotherm of the stable γ INM form. Thermograms of all the SD formulations characterized showed the complete disappearance of the melting endotherm, indicating that INM was completely converted to its amorphous state, SD formulations existed as a two-phase system due to the presence of the semi-crystalline P407. 

In the low-temperature region of the DSC scan, the glass transition temperature [*T*_g_] of aINM was detected at 45.5 °C [2]. Pezzoli et al. (2019) also reported a *T*_g_ of 45.5 °C for aINM when processed using HME [2]. PVP VA64/PL-S630 were indeed amorphous with a *T*_g_ at 109 and 107 °C respectively, as expected, based on the XRPD results. 

A separate *T*_g_ attributable to INM was not present in the hyper DSC thermograms of the SD formulations, suggesting a complete absence of a separated phase rich in amorphous INM and a good level of mixing. P407 also displayed an endothermic peak at 58.16 °C due to its semi-crystalline structure with an enthalpy of fusion (Δ*H*_m_) of 117.1 J/g which corresponds to its crystalline fraction. Therefore, assuming that 100% semi-crystalline P407 has an enthalpy of fusion of 117.1 J/g, the degree of crystallinity of P407 within SD formulations was calculated. The P407 enthalpy of fusion (J/g) was calculated by dividing the area of the endothermic (melt) peak by the heating rate, which was 100°C/min. The % crystallinity of each of the SD formulations was calculated by dividing the P407 enthalpy of fusion (J/g) of each of the formulations by the theoretical enthalpy of fusion (J/g) of both the 5% and 15% P407 formulations. 

For SD1-AC to SD6-AC, the P407 enthalpy of fusion (J/g) in Table 7 was divided by 5.86 J/g for the 5% P407 formulations and 17.56 J/g for the 15% P407 formulations and multiplied by 100 to determine the % crystallinity in each of the formulations. For example, for SD1-AC, 0.593 J/g was divided by 5.86 J/g and multiplied by 100 to give 10.12 % P407 crystallinity. Table 7 displays an overview of the degree of crystallinity exhibited by the various extrudates and glass transition temperatures (experimental vs predicted) relative to the pure crystalline drug. After careful analysis of the hyper DSC thermograms of the SD formulations, the endotherm attributed to the melting of the P407 fraction was detected in all formulations at 52.63 °C, with a mean ΔH_m_ of 0.862 J/g for the 5% P407 SD formulations and 6.68 J/g for the 15% P407 SD formulations. When calculated, ΔH_m_ of 100% crystalline P407 in 5% SD formulations was 5.86 J/g which corresponds to 14.7% crystalline P407. Similarly, 15% SD formulations contain in theory 38.0% P407.

Considering the concentration of plasticizer present in the amorphous SD formulations, it was estimated that the % crystallinity present in the 5% P407 formulations was within the range of 10.12% to 17.61% in its crystalline form and 27.33% to 54.39% for the 15% P407 formulations. It is important to note that the formulations with the higher P407 loadings had a degree of crystallinity that was significantly higher than formulations with low P407 loading since the APIs can be incorporated into the core of P407 micelle at high loads [24]. 

It is important that cooling also had a significant effect on the degree of crystallinity. For example, SD2-NT had the highest % crystallinity, indicating that using a high P407 loading with a slow cooling process increases the % crystallinity. Whereas SD2-Liq N_2_ which was a rapid cooling method, had a low % crystallinity. 

For all SD formulations, a single *T*_g_ was observed which confirms that INM was converted to its amorphous form, indicating that the polymers and drug were present in a two-phase system as P407 was still semi-crystalline (see Figure 8 and Figure 9). 

The experimental glass transition temperatures were significantly different to the predicted *T*_g_s calculated using the Couchman–Karasz equation, as shown in Table 7, as drug–polymer loading can have a significant effect on the *T*_g_. The effect of drug and polymer loading on the glass transition of SD formulations was predicted using the model reported by Couchman and Karasz [25,26]. The theoretical glass transition of the SD formulations was calculated using the following Equation (5):

(5) $${Tg}_{\mathrm{mix}\;(\mathrm{HME}\;\mathrm{System})}=\frac{\left(x_{1}\Delta C_{P1}{Tg}_{1}+x_{2}\Delta C_{P2}{Tg}_{2}\right)}{\left(x_{1}\Delta C_{P1}+x_{2}\Delta C_{P2}\right)}$$

where *T_g_*_1_ and *T_g_*_2_, are the *T*_g_s of API and polymeric carriers, respectively, *X*_1_ and *X*_2_ correspond to the weight fractions of each of the pure materials and Δ*C*p1 and Δ*C*p2 corresponds to the change in the specific heat capacity at each of the glass transition temperature, respectively [27].

This analysis confirmed that P407 was not solubilized and is present in its semi-crystalline form with some level of compatibility with PL-S630/PVP VA64-INM system due to the reduction of the enthalpy of fusion and melting temperature in the various SD formulations. This was consistent with higher Bragg peak intensities observed in the XRPD data from the 15% P407 sample with 30% drug loading. 

The cooling method used had very little effect on the *T_g_*. However, the degree of crystallinity was also affected by (1) the cooling method used (2) % P407 loading and (3) the polymeric carrier used. For example, SD4 had a higher % crystallinity compared to SD6. This is because PL-S630 and PVP VA64 possess different solubility properties with PVP VA64-INM SD formulations having a significantly greater than INM-PL-S630 SDs. There was a significant difference in the *T_g_*s between SD4 and SD6 due to the different solid-state properties of PL-S630 and PVP VA64. However, the inclusion of P407 improved the processability and fragility of the extrudates, and also lowered the processing temperatures. 

The HSPs alongside XRPD and hyper DSC confirmed that the drug and polymer were indeed miscible. XRPD and hyper DSC confirmed successful conversion of INM to the amorphous form as mentioned before, hyper DSC also confirmed that although P407 remained semi-crystalline, as only one single *T_g_* was present in all SD formulations. According to Couchman and Kasasz, (1978) if a SD formulation only contains one *T_g_* then the drug and polymer are indeed miscible. ATR-FTIR and Raman spectroscopy confirmed hydrogen bonding interaction between PVP VA64/PL-S630 and INM due to the interaction between the amide carbonyl C=O of the polymer and the free carboxylic acid C=O of INM. For successful conversion to the amorphous form interaction between drug and polymer is necessary. 

### 3.2. Solubility Studies

#### 3.2.1. Phase Solubility Studies

It is well reported in literature that the API INM due to it being a weak acid (pKa 4.5) (INM can partially dissociate into ions within aqueous solution). Its solubility is affected by pH [28], meaning that the solubility of INM increases with increasing polymer concentration and pH. In this work, phase solubility studies were carried out in buffer of pH 1.2. PL-S630 and PVP VA64 are non-ionic polymeric carriers and display a pH-independent solubility. The INM aqueous solubility from the several ternary and quaternary SD formulations are shown in Table 8. For the SD formulations, after 24 h the quaternary SD formulations had the highest aqueous solubility with a value of 34.17 µg/mL (Quart SD1) with 30% INM loading as shown in Table 8. The SD formulations after 3 h had a higher solubility for INM compared to the physical mixtures. This may have been due to the conversion to the amorphous form, drug–polymer interaction and drug–polymer miscibility. Crystalline INM is present in the physical mixtures resulting in recrystallization. 

After 24 h, the physical mixtures had a significantly higher solubility as polymeric particles hydrated rapidly resulting in the increased wettability of the drug particles [5,29]. However, this depends on the % composition of drug and polymer used as shown in Table 8. The kinetic solubility of the mixed copovidone systems was considerably higher than the ternary systems (Table 4) possibly because of the greater shift in the vinyl acetate carbonyl of PVP VA64/PL-S630 due to hydrogen bonding as shown in Figure 5. However higher shifts do not guarantee a higher kinetic solubility. 

This is true when the SD comes into contact with water causing a change in the kinetics and thermodynamics of the system. The decrease in INM solubility was due to recrystallization of the API. The cooling method used also had a significant effect on the solubility of INM. For example, SD2-NT had a significantly higher solubility than SD2-AC and SD2-Liq N_2_. 

The effect of cooling on the SD formulations with PVP VA64 and PL-S630 had a significant effect on the dissolution properties. After 24 h, the SD formulation cooled to normal room temperature (NT) and air-cooled formulations had the highest aqueous solubility of INM as slow cooling of aINM increases physical stability as reported by Baghel et al. (2015) [11]. As SD2-NT had the highest degree of crystallinity due to the slow cooling process, it increased the solubility of INM significantly. 

There was also a significant improvement in the kinetic solubility of INM after 24 h with a maximum kinetic solubility of 34.17 µg/mL, compared to 30 µg/mL after 72 h reported by Chokshi et al. (2008) [10]. SD2-NT had the highest solubility with a value of 34.17 µg/mL after 24 h, which again shows that slow cooling increases solubility. Like the degree of crystallinity, there was a significant difference between SD4 and SD6 due to different solubility properties of PVP VA64 and PL-S630, as mentioned previously. 

#### 3.2.2. In-Vitro Dissolution Studies 

The in vitro dissolution rates of INM and selected SD formulations, shown in Table 9 and Table 10, were performed using non-sink conditions in pH buffer 1.2. Table 9 shows the concentration of INM (µg/mL) at various time intervals over 3 h. Control of the particle size of each formulation was vital in this study, as it can affect dynamic solubility of each formulation. The particle size of each formulation was 200 microns to ensure uniformity. The formulation equal to 100 mg of INM was placed within the dissolution vessels. 

It is important to note that there was no interference from the polymers which therefore did not affect the value of UV absorption. The λ max for both PL-S630 and PVP VA64 is 440 nm for vinyl-pyrrolidone and 258 nm for vinyl acetate as stated in the literature [30,31]. INM has a λ max of 320 nm. 

**Figure 10:**
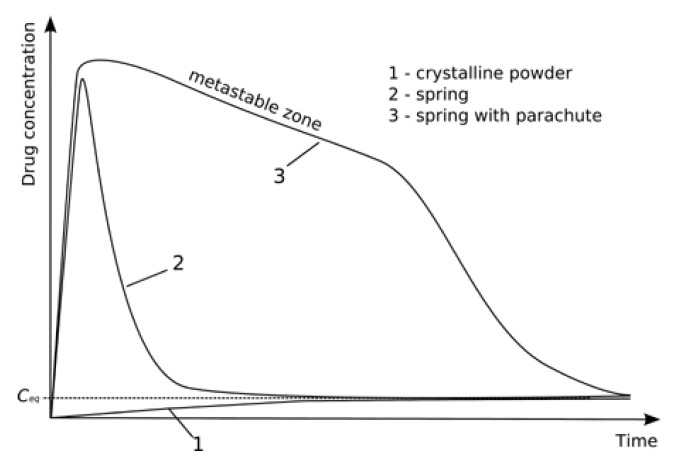
Illustration of non-sink conditions used in the dissolution test. Dissolution profile 1: dissolution of the crystalline API; profile 2: dissolution of a “spring” effect of the API without the presence of dissolution enhancers profile 3: dissolution of a “spring” and ‘’parachute’’ form of the API in the presence of dissolution enhancers act as a “parachute.” *C*_eq_ represents equilibrium solubility.

The aqueous solubility of crystalline and amorphous INM was 1.2 and 2.4 µg/mL respectively [5]. Non-sink conditions were used for this study as crystallization of APIs and nucleation within supersaturated dissolution conditions can be observed [32]. An illustration of non-sink conditions is shown in Figure 10. In Figure 10, the SD has no initial crystallinity due to the conversion to the amorphous state, but several crystals grow during the dissolution test as the supersaturation generated results in phase separation and nucleation [33]. The dissolution of dissolved INM depended upon the dissolution rate comparative to the rate of crystallization. If the dissolution rate is rapid relative to crystallization, then 100% release may be achieved, with a subsequent decrease in the solution concentration as crystallization occurs (shown in Figure 10). If the rate of crystallization is rapid compared to the dissolution rate, then 100% drug release will not be achieved and will display a ‘’spring’’ and ‘’parachute’’ effect which is the case in this study. 

**Figure 11:**
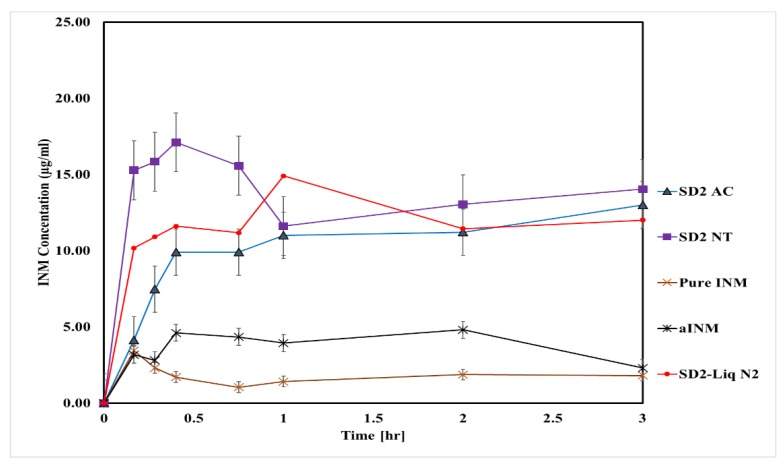
In-vitro dissolution profiles of air-cooled SD formulations performed in buffer of pH 1.2.

**Figure 12:**
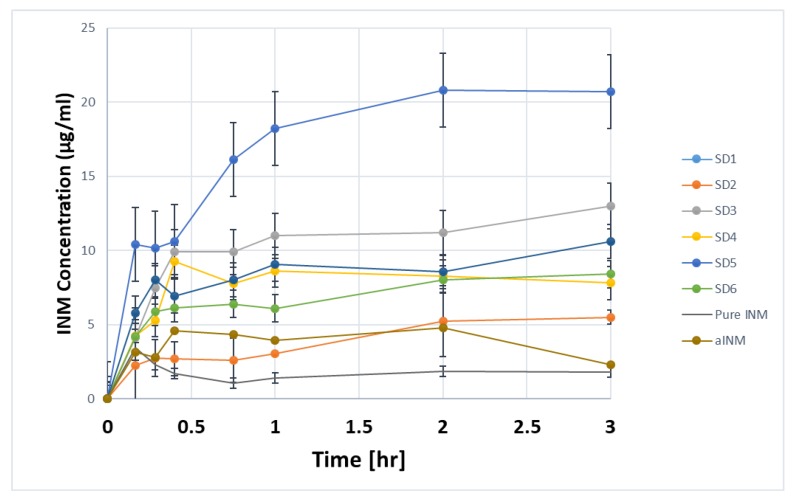
In-vitro dissolution profiles of quaternary SD formulations performed in buffer of pH 1.2. The maximum aqueous solubility is 20.73 µg/mL after 3 h. (AC = Air cooled, NT = Normal room temperature and Liq N_2_ = Liquid Nitrogen).

The dissolution profiles for melt extrudates containing pure drug and polymers are shown in Figure 11 and Figure 12. The aqueous solubility of dissolved INM in SD formulations increased compared to aINM and pure INM respectively. Pure and aINM had an aqueous solubility of 1.2 µg/mL and 2.4 µg/mL as expected, which is similar to the values reported in the literature [28]. Amorphous INM had a kinetic solubility of 2.4 µg/mL as expected due to the transformation to the amorphous state. The increase in the aqueous solubility was dependent upon the concentration of polymer used. 

In this study there was a tendency that as the concentration of P407 increased, the drug concentration also increased (Figure 12). The aqueous solubility of dissolved INM significantly increased compared to the values reported in literature. Chokshi et al. (2008) [10] prepared INM-PVP VA64 binary mixtures and recorded a kinetic solubility of 10 µg/mL and 30 µg/mL in pH buffer 1.2 after 24 and 72 h using 30% INM. 

Table 10 shows the SP of INM after 3 h in pH buffer 1.2 under non-sink conditions. For the purposes of this work, only the supersaturation parameter (SP) of SD formulations after 3 h was selected and calculated. 

According to Chen et al. (2015), SP apparently is a dimensionless parameter with a range between zero and one. Zero means no supersaturation power, while one indicates the tendency of drug precipitation to occur is reduced [34]. SP is a function of the chemical structure of the drug, initial drug concentration, type of polymer and concentration of polymer. 

Table 10 shows that all formulations had a SP value of 0.24 or above indicating that the INM was resilient against drug precipitation, however the quaternary formulation SD1 contained the highest SP value indication that polymer type and concentration of polymer had a significant effect on the ability of the SD to inhibit drug precipitation and recrystallization. 

During the extrusion process, INM was transformed to the amorphous state in all SD formulations. Therefore, as a result, the aqueous solubility of INM was significantly greater compared to both amorphous and crystalline INM with a maximum aqueous solubility of 20.73 µg/mL. This study also showed that the cooling method used for extrusion had a significant impact on the kinetic solubility of INM. The highest aqueous solubility observed was 34.00 µg/mL for SD2 NT containing 30% INM after 24 h. The dissolution concentration increased by 10 times as compared to pure and amorphous drug. 

All SD formulations with highest P407 loading (15%) had a significantly higher solubility compared to the 5% P407 formulations after 3 h. The significant increase in aqueous solubility was related to the degree of crystallinity, cooling process used and interaction between polymeric carrier and API, including polymer–drug miscibility. The P407 micelle core contains propylene oxide which is hydrophobic which when incorporated into INM resulted in increased solubility of INM. P407 in nature is a unimer which when placed into solution can form micelles via self-assembly [35]. 

According to Suksiriworapong et al. (2013) the critical micelle concentration of P407 is 2.8 × 10^−8^ M (0.03 µg/mL), as the concentration of drug and SD formulations in this study was greater than 0.03 µg/mL it resulted in micelle formation as shown in Table 4 [36]. Moreover, the degree of crystallinity was significantly lower in the higher P407 loadings, increasing solubility. P407 can result in many benefits such as greater wetting, increasing surface area and decreasing interfacial tension between API and the dissolution medium. It is stated in the literature that reducing the interfacial tension decreases nucleation [37]. 

The recrystallization of amorphous INM in solid solutions was dependent upon three factors; % P407 loading, degree of P407 crystallinity and cooling method used. It is well reported in literature that high P407 loading can lead to recrystallization due to the hydrophobic P407 propylene oxide core [35]. Slow cooling is ideal for a process that does not require high energy removal. It is less expensive regarding extrusion, it is easier to maintain, has lower operating costs compared to liquid nitrogen and requires less space compared to fluid cooling. 

**Figure 13:**
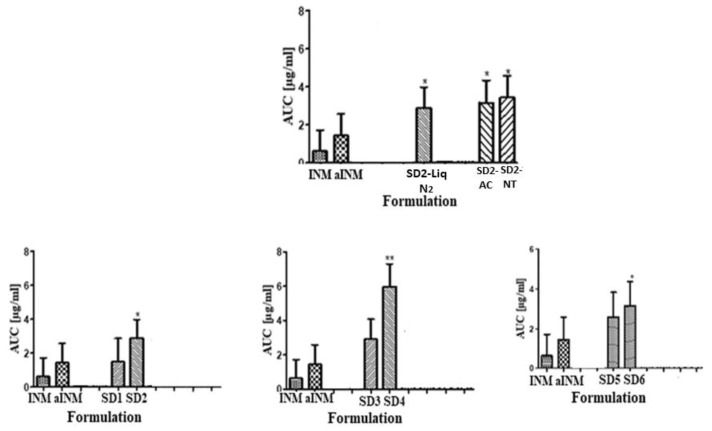
Graphical representations of AUC of quaternary and ternary SD formulations in pH 1.2. ** and * represents the statistical difference (*p* < 0.05) between ASD, amorphous INM and pure INM respectively, for the one-way ANOVA and Tukey–Kramer post hoc test. (AC = Air cooled, NT = Normal room temperature and Liq N_2_ = Liquid Nitrogen).

The AUC of pure and aINM was compared to the AUC of each SD formulation using statistical analysis. The statistical tests used were Tukey Kramer post hoc test and 1-way ANOVA (Figure 13). For both the quaternary and ternary SD formulations with the exception of SD2, SD4, and SD6 there was no statistical difference between amorphous and crystalline INM. The overall effect of drug, polymer concentration and % wt. of P407 did have a significant effect on the solubility of INM and AUC in solution. 

Figure 13 shows that the formulations containing the highest P407 loading were statistically different to crystalline INM which shows that the increase in P407 increased the solubility of INM [5]. Figure 13 also shows that cooling had a significant effect on solubility. SD2-NT had the highest solubility after 3 and 24 h with values of 14.03 µg/mL and 34.00 µg/mL. Whereas the rapid cooling method SD2-Liq N_2_ had a solubility of 12.00 µg/mL and 20.00 µg/mL respectively. This corresponds to the values reported for the degree of crystallinity, with SD2-NT having the highest degree of crystallinity. This shows that the greater the % crystallinity of P407 (see Table 7), the greater the solubility. SD2-NT had the highest aqueous solubility due to the slow cooling process. Therefore, cooling influenced the solubility of INM and AUC in solution. 

The solubility also depended upon the polymeric carrier used as mentioned already. There was a significant difference in solubility between SD4 and SD6 which contain PVP VA64 and PL-S630 respectively. SD4 had a kinetic solubility of 20.73 µg/mL compared to 10.60 µg/mL for SD6. It is important to note that the different solid-state and dissolution properties of PVP VA64 and PL-S630 significantly affect the solubility of INM achieved. 

### 3.3. Accelerated Stability Studies

**Figure 14:**
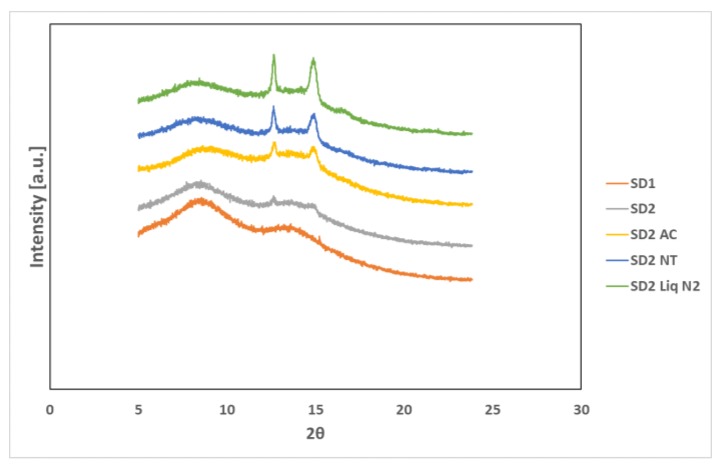
XRPD diffractograms of SD formulations and XRPD diffractograms of the SD formulation used to investigate the effect of cooling after 5 months of a stability study under accelerated conditions of 40 °C and 75% RH. P407 was still present in its crystalline form in all formulations (AC = Air cooled, NT = Normal room temperature and Liq N_2_ = Liquid Nitrogen).

**Figure 15:**
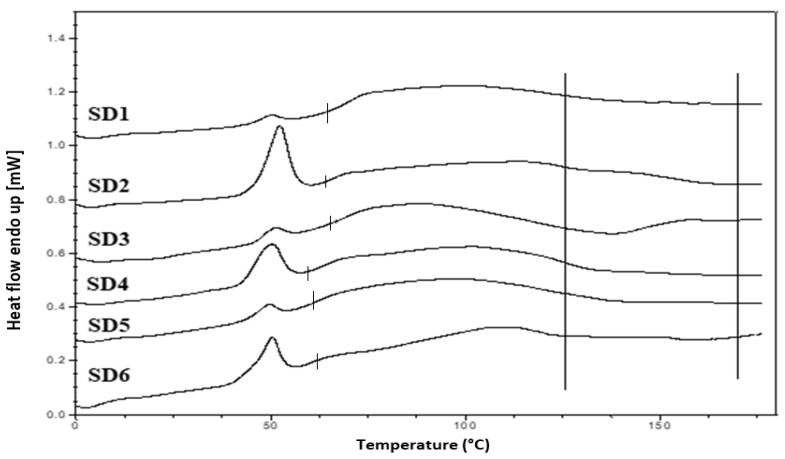
Hyper DSC thermograms of air-cooled SD formulations after 5 months of a stability study under accelerated conditions of 40 °C and 75% RH. P407 was still present in its crystalline form in all formulations. The area of interest for crystalline INM melting endotherm is marked by straight lines. *T*_g_ is indicated by a tick.

**Figure 16:**
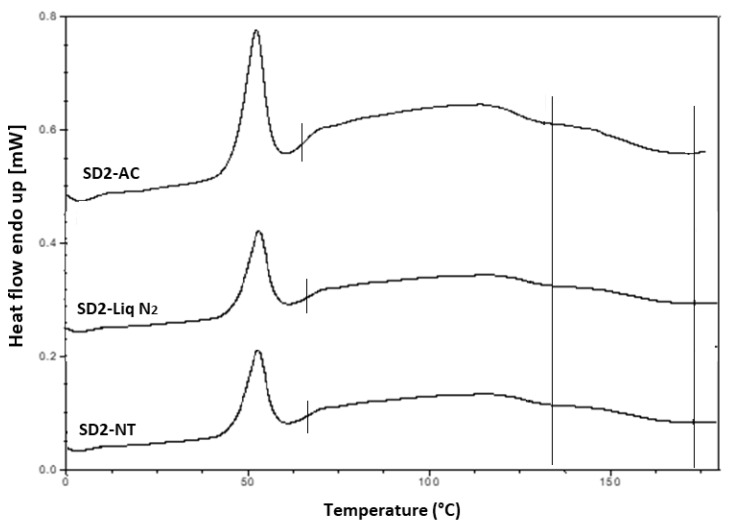
Hyper DSC thermograms of selected SD formulations used to investigate the effect of cooling after 5 months of a stability study under accelerated conditions of 40 °C and 75% RH. P407 is was still present in its crystalline form in all formulations (AC = Air cooled, NT = Normal room temperature and Liq N_2_ = Liquid Nitrogen). The area of interest for crystalline INM melting endotherm is marked by straight lines. *T*_g_ is indicated by a tick.

Accelerated stability studies were performed at 40 °C/75% RH using the Amebis stability testing system. After 5 months it was noted that some samples had adsorbed significant quantities of water and that all samples had attained a darker yellow colour. Samples were removed from the humidity chambers and dried overnight under vacuum to remove adsorbed water. This was because water acts as a plasticizer, thereby enhancing the molecular mobility of drugs and polymers [38]. They were dried for 24h in a desiccator over phosphorous pentoxide. XRPD and hyper DSC confirmed that after 5 months INM remained amorphous for all SD formulations (Figure 14, Figure 15 and Figure 16). The Bragg peaks associated with crystalline INM were completely absent in all XRPD diffractograms of SDs after 5 months. 

In the XRPD diffractograms shown in Figure 14 after 5 months stability, the Bragg peaks associated with crystalline INM were completely absent. Hyper DSC also confirmed that INM was stable after 5 months stability studies. For hyper DSC the samples were dried as the moisture was coming off at the same temperature that the *T*_g_ occurred as reported by Hurley et al. (2018) [5]. After 5 months stability, all SD formulations remained amorphous and there was no reduction in *T*_g_ due to moisture removal. Also, there was no significant difference in *T*_g_ prior to and after 5 months stability (See Figure 14, Figure 15 and Figure 16).

This was also shown by Hurley et al. (2018), [5], who prepared SDs using INM as a model drug. All SDs remained amorphous as a result of conversion from the crystalline to amorphous state and as a result of the high molar attraction constant of INM, hydrogen bonding and drug–polymer miscibility. This shows that all ASD formulations are miscible as predicted by the HSPs in Table 1. The results also confirm that the cooling method used did not have a significant impact on the amorphous stability and *T*_g_ of INM. 

## 4. Conclusions

The HSPs, alongside XRPD and hyper DSC, confirmed that the drug and polymer were indeed miscible. XRPD and hyper DSC confirmed successful conversion of INM to the amorphous form as mentioned before, hyper DSC also confirmed that although P407 remained semi-crystalline, as only one single *T_g_* was present in all the SD formulations. According to Couchman and Kasasz, (1978) if a SD formulation only contains one *T_g_* then the drug and polymer are indeed miscible. ATR-FTIR and Raman spectroscopy confirmed hydrogen bonding interaction between PVP VA64/PL-S630 and INM due to the interaction between the amide carbonyl C=O of the polymer and the free carboxylic acid C=O of INM. For successful conversion to the amorphous form interaction between drug and polymer is necessary. ATR-FTIR spectroscopy also confirmed that P407 does not interact with INM at a molecular level.

The cooling method used did not have any effect on the *T_g_* when air cooled, cooled to normal room temperature and cooled via liquid nitrogen. However, cooling and the type of polymeric carrier used had a significant effect on the degree of crystallinity and solubility of INM. There was a significant difference in the enthalpy of fusion in the formulation chosen to investigate the effect of cooling. It is also important to note that in the formulations with the higher P407 loadings, the degree of crystallinity was within the range of 27.33% to 54.39%, compared to 10.12% to 17.61% for the 5% SD formulations.

Solubility studies of INM showed a significant increase in the kinetic solubility of INM compared to crystalline INM at 37 °C in pH 1.2. The SD formulations showed a significantly higher dissolution rate of 20.63 µg/mL. The XRPD and hyper stability data showed that the cooling method used did not have a significant effect on the amorphous stability of INM as all SD formulations remained amorphous after 5 months stability testing. 

The results of the hyper DSC and solubility studies show that the greater the % crystallinity of P407, the greater the solubility. However, the cooling process used, significantly affected the degree of crystallinity and solubility also as SD2-NT had the highest aqueous solubility for dissolved INM and the highest crystallinity due to the slow cooling process. Therefore, slow cooling enhances physical stability and solubility, as reported by Baghel et al. (2015) [11].

This work illustrates the significance of examining the cooling method used in order to improve the aqueous solubility, amorphous stability and solid-state properties of BCS class II drugs. 

## Figures and Tables

**Table 1:** Drug/carrier percentages of the HME processed formulations.

Batch identifier No	Composition (% *w*/*w*)			
	PL-S630 (% *w*/*w*)	PVP VA64 (% *w*/*w*)	P407 (% *w*/*w*)	INM (% *w*/*w*)
SD1	32.5	32.5	5	30
SD2	27.5	27.5	15	30
SD3	-	65	5	30
SD4	-	55	15	30
SD5	65	-	5	30
SD6	55	-	15	30

**Table 2:** Calculated solubility parameters using Hansen solubility parameters (δ) and drug–polymer interaction factor (χ).

Compound	δ_t_ (MPa^1/2^)	Δδ (MPa^1/2^)	χ
INM	23.00	-	-
PVP VA64	26.40 ^a^	3.40^a^	0.46
PL-S630	26.40	3.40	0.46
P407	25.50^b^	2.50^b^	0.36

^a^ and ^b^ are values obtained from a previously published report [5].

**Table 3:** Calculation of HSPs and molar volume for INM according to the Hoftyzer-Van Krevelen method.

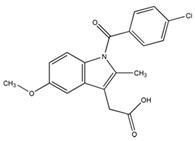					
Group	Frequency	F_di_ (J^1/2^_cm_^3/2^_mol_^−1^)	F^2^_Pi_ (J^1/2^_cm_^3/2^_mol_^−1^)	F_hi_ (J/mol)	Vm ^a^ (cm^3^/mol) (*V*o)
–CH_3_	2	840	0	0	67
–CH_2_–	1	270	0	0	32.2
=CH–	3	600	0	0	40.5
>C=	5	350	0	0	−27.5
Phenylene (o,m,p)	1	1270	12,100	0	52.4
–Cl	1	450	302,500	400	24
–O–	1	100	160,000	3000	3.8
–CO–	1	290	770	2000	10.8
–COOH	1	530	176,400	10,000	28.5
–N<	1	20	640,000	5000	−9
Ring closure 5 or more atoms	2	380	0	0	32
Conjugation in ring for each double bond	4	0	0	0	-8.8
∑		5100	1291770	27400	254.7

**Table 4:** Calculation of HSPs and molar volume for PVP VA64/PL-S630 according to the Hoftyzer-Van Krevelen method.

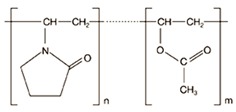					
Group	Frequency	F_di_ (J^1/2^_cm_^3/2^_mol_^−1^)	F^2^_Pi_ (J^1/2^_cm_^3/2^_mol_^−1^)	F_hi_ (J/mol)	Vm ^a^ (cm^3^/mol) (*V*o)
–CH_3_	1	420	0	0	33.5
–CH_2_–	2	540	0	0	32.2
–CH–	2	160	0	0	−2.00
–CO–	1	290	770	2000	10.8
–N<	1	20.0	800	5000	-9.00
–COO–	1	390	490	7000	18.0
Ring 5 or more	1	190	0	0	16.0
∑		2010.0	1,473,000	14,000	99.50

**Table 5:** Calculation of HSPs and molar volume for P407 according to the Hoftyzer-Van Krevelen method.

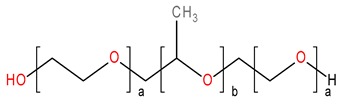					
Group	Frequency	F_di_ (J^1/2^_cm_^3/2^_mol_^−1^)	F^2^_Pi_ (J^1/2^_cm_^3/2^_mol_^−1^)	F_hi_ (J/mol)	Vm ^a^ (cm^3^/mol) (*V*o)
–CH_3_	1	420	0	0	33.5
–CH_2_–	5	2100	0	0	80.5
–OH	2	420	500,000	20,000	20
–O–	2	200	320,000	3000	7.6
∑		2390	820,000	46,000	141.6

**Table 6:** Calculated HSPs of and drug–polymer interaction factor for drug and polymers.

Identifier	Solubility Parameters						
	δ_d_ (MPa^1/2^)	δ_p_ (MPa^1/2^)	δ_h_ (MPa^1/2^)	δ_v_ (MPa^1/2^) (Vo)	δ_total_ (MPa^1/2^)	Δδ_t_ (MPa^1/2^)	χ
**INM**	20.02	4.46	10.37	20.51	23.00	-	-
**PVP VA64**	20.20	12.20	11.86	23.59	26.40	3.40	0.46
**PL-S630**	20.20	12.20	11.86	23.59	26.40	3.40	0.46
**P407**	16.88	6.40	18.02	18.05	25.50	2.50	0.36

**Table 7:** Experimental *T*_g_, Predicted *T*_g_, P407 enthalpy of fusion (J/g) and % crystallinity of various SD formulations and selected SD formulations used to investigate the effect of cooling on the solubility of INM.

Identifier No	Experimental Tg	Predicted Tg (Couchman-Karasz)	P407 Enthalpy of fusion (J/g)	% Crystallinity
**Crystalline INM**	45.56	-	-	-
**P407**	−67.00	-	117.1	-
**SD1-AC**	74.91	83.54	0.593	10.12
**SD2-AC**	67.42	84.70	9.551	54.39
**SD3-AC**	71.82	80.14	0.963	16.43
**SD4-AC**	60.54	78.26	5.697	32.44
**SD5-AC**	74.58	86.94	1.032	17.61
**SD6-AC**	67.42	84.71	4.799	27.33
**SD2-NT**	69.24	84.70	11.90	67.78
**SD2-Liq N_2_**	70.19	84.70	9.478	53.97

**Table 8:** Comparison of the aqueous solubility of INM and standard deviation (STD) from various quaternary and ternary SD formulations and physical blends in pH buffer 1.2 after 3 and 24 h respectively. Physical blends contain crystalline INM.

Identifier No.	Solubility of INM (µg/mL) after 3 h				Solubility of INM (µg/mL) after 24 h			
	SD	STD	Physical Blend	STD	Physical Blend	STD	SD	STD
SD1	5.50	0.40	3.27	0.03	26.13	0.01	23.47	0.03
SD2	13.00	0.15	3.30	0.00	27.30	0.02	34.17	0.00
SD3	7.80	0.69	2.74	0.00	21.93	0.00	4.00	0.00
SD4	20.73	0.65	2.59	0.01	26.50	0.01	6.13	0.01
SD5	8.40	1.20	0.97	0.01	7.77	0.01	6.43	0.01
SD6	10.60	1.20	1.47	0.01	11.57	0.015	10.60	0.01
SD2-AC	13.00	0.15	3.03	0.00	24.30	0.03	32.17	0.00
SD2-NT	14.03	0.26	4.03	0.01	27.30	0.02	34.00	0.01
SD2-Liq N_2_	12.00	1.35	2.03	0.00	15.12	0.04	20.00	0.00

**Table 9:** Comparison of the aqueous solubility of INM from various quaternary and ternary SD formulations in pH buffer 1.2 taken at various time intervals over 24 h.

Identifier	Con. of INM (µg/mL) at 0 h	Conc. of INM (µg/mL) at 0.2 h	Conc. of INM (µg/mL) at 0.3 h	Conc. of INM (µg/mL) at 0.4 h	Conc. Of INM (µg/mL) at 0.75 h	Conc. of INM (µg/mL) at 1 h	Conc. of INM (µg/mL) at 2 h	Conc. of INM (µg/mL) at 3 h
SD1	0.00	2.27	2.73	2.70	2.60	3.07	5.23	5.50
SD2	0.00	4.13	7.46	9.90	9.90	11.00	11.20	13.00
SD3	0.00	4.23	5.30	9.27	7.77	8.63	8.27	7.80
SD4	0.00	10.40	10.17	10.63	16.13	10.80	20.83	20.73
SD5	0.00	4.17	5.87	6.13	6.40	6.10	8.03	8.40
SD6	0.00	5.80	8.00	6.93	8.03	9.07	8.57	10.60
SD2-AC	0.00	4.13	7.46	9.90	9.90	11.00	11.20	13.00
SD2-NT	0.00	15.27	15.83	17.10	15.57	11.60	13.03	14.03
SD2-Liq N_2_	0.00	10.17	10.90	11.60	11.17	14.90	11.43	12.00

**Table 10:** Comparison of the aqueous solubility of INM from various quaternary and ternary SD formulations in pH buffer 1.2 after 3 and 24 h. Supersaturation parameter [SP] was calculated for SD formulations after 3 h.

Identifier No.	No. of Phases	Conc. of INM, 3 h. (µg/mL)	STD	Conc. of INM, 24 h. (µg/mL)	STD	SP of INM after 3 h
SD1	2	5.50	0.40	23.47	0.01	0.97
SD2	2	13.00	0.15	34.17	0.02	0.98
SD3	2	7.80	0.69	4.00	0.00	0.49
SD4	2	20.73	0.65	6.13	0.01	0.24
SD5	2	8.40	1.20	6.43	0.01	0.56
SD6	2	10.60	1.20	10.60	0.015	0.45
SD2-AC	2	13.00	0.15	32.17	0.03	0.49
SD2-NT	2	14.03	0.26	34.00	0.02	0.70
SD2-Liq N2	2	12.00	1.35	20.00	0.04	0.30
